# Rare Manifestation of a c.290 C>T, p.Gly97Glu *VCP* Mutation

**DOI:** 10.1155/2015/239167

**Published:** 2015-03-23

**Authors:** Nivedita U. Jerath, Cameron D. Crockett, Steven A. Moore, Michael E. Shy, Conrad C. Weihl, Tsui-Fen Chou, Tiffany Grider, Michael A. Gonzalez, Stephan Zuchner, Andrea Swenson

**Affiliations:** ^1^Department of Neurology, Carver College of Medicine, University of Iowa, Iowa City, IA 52242, USA; ^2^Department of Pathology, Carver College of Medicine, University of Iowa, Iowa City, IA 52242, USA; ^3^Department of Neurology, Washington University School of Medicine, St. Louis, MO 63110, USA; ^4^Division of Medical Genetics, Department of Pediatrics, Harbor-UCLA Medical Centre, Los Angeles Biomedical Research Institute, Torrance, CA 90502, USA; ^5^Dr. John T. Macdonald Foundation Department of Human Genetics and John P. Hussman Institute for Human Genomics, University of Miami Miller School of Medicine, 1501 NW 10 Avenue, Miami, FL 33136, USA

## Abstract

*Introduction*. The valosin-containing protein (VCP) regulates several distinct cellular processes. Consistent with this, *VCP* mutations manifest variable clinical phenotypes among and within families and are a diagnostic challenge. *Methods*. A 60-year-old man who played ice hockey into his 50's was evaluated by electrodiagnostics, muscle biopsy, and molecular genetics. *Results*. With long-standing pes cavus and toe walking, our patient developed progressive weakness, cramps, memory loss, and paresthesias at age 52. An axonal sensorimotor neuropathy was found upon repeated testing at age 58. Neuropathic histopathology was present in the quadriceps, and exome sequencing revealed the *VCP* mutation c.290 C>T, p.Gly97Glu. *Conclusions*. Our patient reflects the clinical heterogeneity of VCP mutations, as his neurological localization is a spectrum between a lower motor neuron disorder and a hereditary axonal peripheral neuropathy such as CMT2. Our case demonstrates a rare manifestation of the c.290 C>T, pGly97Glu *VCP* mutation.

## 1. Introduction

The valosin-containing protein (VCP) has been demonstrated to play a critical role in the maintenance of protein homeostasis through the regulation of protein degradation pathways [1]. Mutations in the gene encoding VCP lead to disruption of autophagy and have been shown to manifest within families as a phenotypically heterogeneous group of presentations including hereditary inclusion body myopathy (IBM), Paget's disease of the bone (PDB), and frontotemporal dementia (FTD), which are known collectively as IBMPFD [2]. More recently, mutations in* VCP *have been implicated as having a role in familial amyotrophic lateral sclerosis (ALS) [3]. Although there is variability in the phenotypic manifestations of* VCP* mutations within families,* VCP*-related disease is inherited in an autosomal dominant manner [2, 3]. Attempts at determining genotype-phenotype correlations for mutations in the* VCP *gene have been unsuccessful, though analysis of the two most common mutations (p.Arg155Cys and p.Arg155His) showed a significant variation in survival between the groups, suggesting that mutation variants may hold clinical relevance for patients [4].

We report a case of a patient presenting for evaluation of progressive lower extremity weakness and paresthesias in the context of family history of ALS, dementia, and PDB. Exome sequencing revealed a previously reported c.290 C>T, p.Gly97Glu mutation in* VCP*; our patient's phenotype is different from the previously reported case of IBMPFD [5]. The clinical presentation along a continuum from an axonal sensorimotor polyneuropathy to a lower motor neuronopathy in this patient expands the known clinical phenotypes of* VCP*-related disease.

## 2. Case Report

The proband is a 60-year-old man of Dutch and Italian descent who was the product of a normal pregnancy and delivery. Although he was a toe walker with high arches and hammertoes, he reached his developmental milestones (including walking) on time. In grade school, he kept up with his peers but was always the slowest runner. He rode a bicycle and played ice hockey until age 52 when he sprained his left ankle and began noticing progressive left greater than right leg weakness. At age 55, he began noticing paresthesias and numbness in his toes with the left side worse than the right. By age 57, holding his trumpet was difficult, although he could still play it well. Three years later, he was unable to climb stairs, had multiple falls, and began to use a walker. He later developed cramps in his hands and trouble with fine finger dexterity. Additionally, he noticed memory and word-finding difficulties.

**Figure 1:**
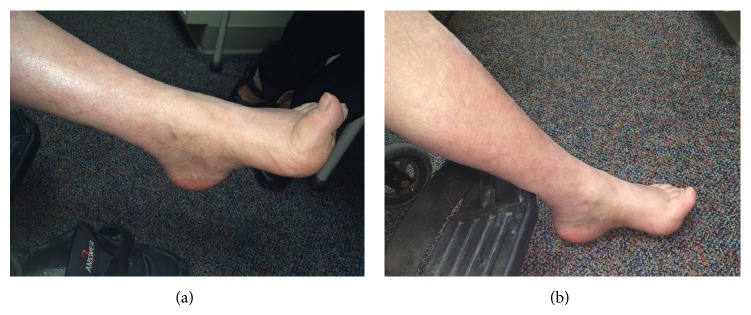
Evidence of pes cavus and hammer toes in our patient.

Upon neurological examination in our clinic at age 60, he presented with bilateral scapular winging, lumbar lordosis, pes cavus, hammer toes, and tight heel cords (Figure 1). Strength exam (MRC scale) demonstrated proximal upper and proximal/distal lower extremity weakness. There was weakness in trapezius (R/L) 3/3 with scapular winging as well as weakness in the iliopsoas 4+/4+, quadriceps 4+/5, knee flexion 4/4, foot dorsiflexion 1/4, and foot plantar flexion 4+/5. Vibratory sense and pinprick were decreased distally in the lower extremities. Deep tendon reflexes were depressed throughout the upper and lower extremities with absent ankle jerks and downgoing toes. There was no tremor. Cerebellar testing was normal. Gait was wide-based with bilateral steppage; he was unable to walk 25 feet independently due to the weakness in his lower extremities. Speech and language were intact. Cranial nerves were normal including facial strength, facial sensation, and tongue strength. No fasciculations were noted on exam. Of note, he did not develop any upper motor neuron signs over the years including hyperreflexia, dysphagia, dysarthria, or dyspnea.

Laboratory testing included creatine kinase level of 930 u/L (normal 40–200 u/L) and a normal alkaline phosphatase level.

### 2.1. Electrodiagnostic Exam

Although the abnormalities on the EMG remained constant, nerve conduction studies (NCS) changed over time.

At age 56, needle EMG revealed fibrillations, positive sharp waves, fasciculations, and large amplitude motor unit potentials with reduced recruitment in bilateral tibialis anterior, bilateral vastus lateralis, left gastrocnemius, left deltoid, left first dorsal interosseous, and left thoracic paraspinal musculature. Nerve conduction studies of the left ulnar, sural, and tibial nerves were normal. At this time, the impression was a lower motor neuron syndrome.

At age 58, although the EMG remained the same, sensory and motor nerve conduction studies were now abnormal. The left median, ulnar, and peroneal motor nerve conduction velocities were normal. Compound muscle action potentials (CMAPs) of the left median, ulnar, and peroneal nerves were normal. Sensory nerve action potentials (SNAPs) of the left median, radial, and superficial peroneal nerves were normal; right ulnar SNAP had reduced amplitude (13 uV). The left sural and superficial peroneal sensory responses were absent; right sural sensory responses had reduced amplitude (4 uV). Left median and ulnar distal motor latencies were prolonged (5.5 ms and 4.2 ms, resp.); left median sensory distal latency was mildly prolonged at 4.0 ms. At this time, the impression was an axonal sensorimotor neuropathy.

The axonal sensorimotor neuropathy was worked up for acquired causes. Laboratory testing included normal thyroid stimulating hormone, serum protein electrophoresis, serum immunofixation, vitamin B-12, and fasting glucose.

### 2.2. Muscle Biopsy

**Figure 2:**
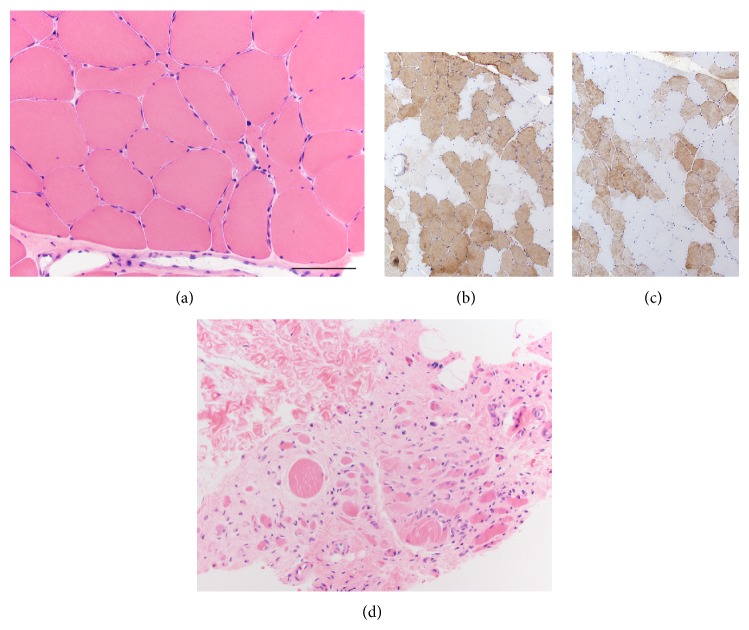
Muscle biopsy of the quadriceps revealed chronic active neurogenic atrophy ((a), H&E). Fiber type grouping is seen in immunoperoxidase staining for both slow myosin (b) and fast myosin (c). Some muscle fascicles are much more severely affected and show features of end stage muscle (d). Scale bar = 100 *μ*m in panels (a) and (d); 150 *μ*m in panels (b) and (c).

Muscle biopsy of the quadriceps at age 60 revealed chronic, active neurogenic atrophy. Paraffin and frozen H&E sections, NADH, and trichrome staining revealed skeletal muscle with moderate variation in fiber size due to the presence of angulated, atrophic fibers in small groups (Figure 2(a)). Immunoperoxidase stains for slow and fast myosin heavy chains revealed small fiber type groups (Figures 2(b) and 2(c)). Rare targetoid fibers were seen (not shown here). Some muscle fascicles had end stage muscle pathology (Figure 2(d)), likely the result of longstanding neuropathic disease. No inflammation, myonecrosis, regeneration, endomysial fibrosis, inclusion bodies, rimmed vacuoles, or ragged-red fibers were identified. All fibers stained positively for cytochrome C oxidase.

### 2.3. Neuropsychological Testing

Neuropsychological testing revealed only mild impairments as he was noted to have high average to superior baseline functioning. He had only mild weakness in speed of information processing, problem solving, fine motor dexterity, speed in visual motor sequencing, rapid cognitive shifting, verbal memory, and short-term memory for simple geometric designs. He had an otherwise normal performance in all other areas (verbal, nonverbal intellectual skills, single word reading, and language). He was diagnosed with cognitive deficits, not otherwise specified.

### 2.4. Family History

**Figure 3:**
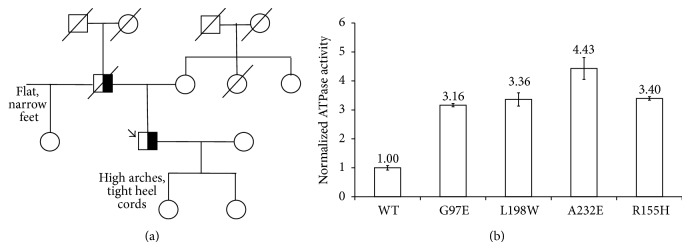
(a) Family Pedigree. Squares indicate men; circles, women; diagonal lines, deceased; black, VCP mutation. An arrow indicates the proband. (b) ATPase activity data that shows that the G97E (Gly97Glu mutation) has increased ATPase activity similar to other mutants. The detailed method was described previously [6]. Purified human p97 proteins (25 nM monomer final concentration) were used in Assay Buffer (50 mM Tris pH 7.4, 20 mM MgCl_2_, 1 mM EDTA, and 0.5 mM TCEP) containing 0.01% Triton X-100 and 200 *μ*M ATP. ATPase activity was determined by addition of Biomol Green Reagent (Enzo Life Sciences). Absorbance at 635 nm was measured after 4 min on the Synergy Neo Microplate Reader (BioTek).

Further investigation into the patient's family history disclosed his father's diagnoses of ALS, dementia, and PDB (Figure 3(a)). Muscle biopsy results from the father are not available. One sister had flat, narrow feet. The patient's teenage daughter has high arches and was a toe walker as a child; she has not had a neurological evaluation and further evaluation has been declined at this time. His other teenage daughter is currently asymptomatic. There is no family history of IBM.

### 2.5. Genetic Testing

Exome sequencing was performed and revealed the* VCP* mutation c.290 C>T, p.Gly97Glu, G97E. This same mutation resulting in the same amino acid substitution has been reported in five family members with PDB. All five affected individuals had varying degrees of muscle weakness diagnosed as inclusion body myopathy, and none have FTD [5].

## 3. Discussion

Phenotypic variability is a hallmark of VCP-related disease. VCP is a member of the type II AAA+ ATPase family mapped to 9p13.3 that is ubiquitously expressed at high levels and exerts an influence on a variety of cellular activities including cell cycle progression, DNA damage repair, the ubiquitin-proteasome system, and autophagic processes [7–9]. This variation in physiological activity allows for an equally impressive range of pathological manifestations to arise from mutations in the* VCP *gene, including IBM, PDB, FTD, and ALS [10]. We believe the* VCP* mutation c.290 C>T, p.Gly97Glu, G97E mutation is pathogenic because the mutation in our patient shows increased ATPase activity, similar to other* VCP* mutations (Figure 3(b)).

**Figure 4:**
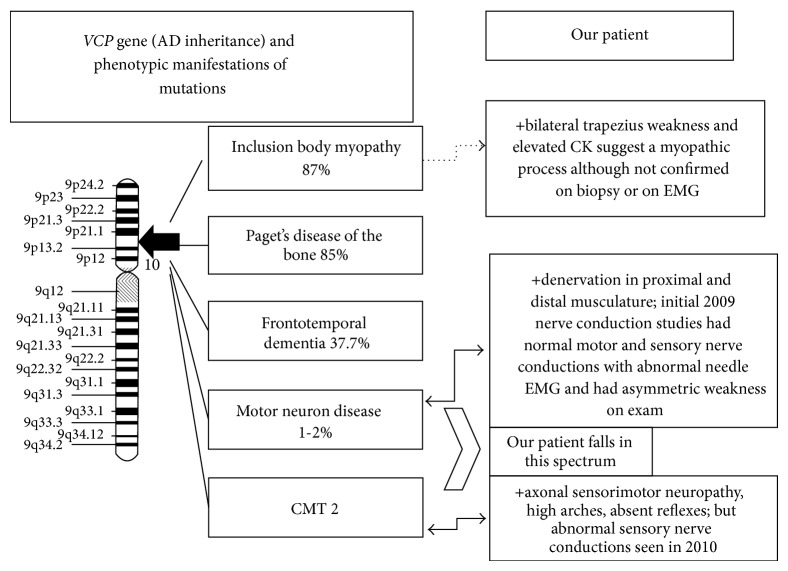
Spectrum of phenotypic manifestations seen in our patient with a* VCP* mutation.

Our patient's presentation is not straightforward clinically. It is not simply a hereditary axonal sensorimotor polyneuropathy (given the asymmetry and bilateral scapular winging), a purely lower motor neuron syndrome (given the bilateral scapular winging and sensory nerve conduction abnormality) nor a myopathy (despite elevated CK and scapular winging, there were no myopathic features on quadriceps muscle biopsy). We propose that our patient presents along a spectrum of a lower motor neuron syndrome and axonal neuropathy (Figure 4). The difficulty in diagnosing a VCP-related condition is due to this extreme heterogeneity in clinical presentations. A recent study demonstrates that a* VCP* mutation can result in CMT 2 further validating the phenotypic variability seen in patients with a* VCP* mutation [11]. Of note, exome sequencing was negative for other genetic variants that are known to cause CMT and a lower motor neuron disease phenotype; this included the* HSPB, BSCL2, GARS, DCTN1, SLC5A7, FBX038, IGHMBP2, ATP7A*, and* SETX* genes.

Of those individuals reported with* VCP *mutations, 87.7% presented with IBM, 45% with PDB, and 37.7% with FTD [12].* VCP *mutations are also estimated to be the cause of 1-2% of familial ALS cases [3]. Further complicating this clinical scenario is the incomplete penetrance of phenotypes of IBM, PDB, and FTD as well as the variability in presentation for* VCP*-related ALS [12, 13]. The Gly97Glu mutation was previously reported in both affected and unaffected members of one family, suggesting that the mutation may not be fully penetrant [5].* VCP *mutations have also been demonstrated to manifest with variable upper motor neuron signs or bulbar findings. EMG results can be neuropathic or myopathic, with many displaying mixed morphology [13]. Other features have been observed less frequently in VCP disease, including dilated cardiomyopathy, cataracts, and axonal sensorimotor polyneuropathy [10].

Because of the variability in clinical presentations, the family history is critical. Mutations in* VCP *are transmitted in an autosomal dominant fashion. Obtaining a pedigree that includes IBM, PDB, FTD, or ALS throughout multiple family members may lead to the consideration of* VCP *sequencing as a diagnostic step. Furthermore, repeated electrodiagnostic testing can be critical to evaluate the progression of the disease. Our case demonstrates how the electrodiagnostic testing changed over the years from the initial electrodiagnostic diagnosis of a lower motor neuron syndrome to the later diagnosis of motor neuron disease plus a sensorimotor axonal neuropathy. Although the neuropathy could be a manifestation of CMT 2 given his long-standing toe walking and pes cavus, the appearance of the neuropathy on electrodiagnostic testing was late. Basic testing for acquired neuropathies was unrevealing, and thus the electrodiagnostic changes could possibly reflect a late onset hereditary neuropathy like CMT 2.

Here we present a case with variable phenotypic manifestations ranging from an axonal sensorimotor polyneuropathy to a lower motor neuron syndrome or even a myopathy all arising from a mutation in the gene encoding* VCP*. This novel presentation further expands the clinical phenotype associated with* VCP *mutations and suggests that sequencing for this gene should be considered for any patients presenting with these symptoms who have family history positive for IBM, PDB, FTD, or ALS.

## References

[1] Ju J.-S., Fuentealba R. A., Miller S. E. (2009). Valosin-containing protein (VCP) is required for autophagy and is disrupted in VCP disease. *The Journal of Cell Biology*.

[2] Watts G. D. J., Wymer J., Kovach M. J. (2004). Inclusion body myopathy associated with Paget disease of bone and frontotemporal dementia is caused by mutant valosin-containing protein. *Nature Genetics*.

[3] Johnson J. O., Mandrioli J., Benatar M. (2010). Exome sequencing reveals VCP mutations as a cause of familial ALS. *Neuron*.

[4] Mehta S. G., Khare M., Ramani R. (2013). Genotype-phenotype studies of VCP-associated inclusion body myopathy with Paget disease of bone and/or frontotemporal dementia. *Clinical Genetics*.

[5] Gu J.-M., Ke Y.-H., Yue H. (2013). A novel VCP mutation as the cause of atypical IBMPFD in a Chinese family. *Bone*.

[6] Chou T.-F., Bulfer S. L., Weihl C. C. (2014). Specific inhibition of p97/VCP ATPase and kinetic analysis demonstrate interaction between D1 and D2 ATPase domains. *Journal of Molecular Biology*.

[7] Bartolome F., Wu H.-C., Burchell V. S. (2013). Pathogenic VCP mutations induce mitochondrial uncoupling and reduced ATP levels. *Neuron*.

[8] Ju J.-S., Weihl C. C. (2010). Inclusion body myopathy, Paget's disease of the bone and fronto-temporal dementia: a disorder of autophagy. *Human Molecular Genetics*.

[9] Iguchi Y., Katsuno M., Ikenaka K., Ishigaki S., Sobue G. (2013). Amyotrophic lateral sclerosis: an update on recent genetic insights. *Journal of Neurology*.

[10] Nalbandian A., Donkervoort S., Dec E. (2011). The multiple faces of valosin-containing protein-associated diseases: inclusion body myopathy with Paget's disease of bone, frontotemporal dementia, and amyotrophic lateral sclerosis. *Journal of Molecular Neuroscience*.

[11] Gonzalez M. A., Feely S. M., Speziani F. (2014). A novel mutation in VCP causes charcot-marie-tooth type 2 disease. *Brain*.

[12] Kimonis V. E., Fulchiero E., Vesa J., Watts G. (2008). VCP disease associated with myopathy, Paget disease of bone and frontotemporal dementia: review of a unique disorder. *Biochimica et Biophysica Acta*.

[13] Benatar M., Wuu J., Fernandez C. (2013). Motor neuron involvement in multisystem proteinopathy: implications for ALS. *Neurology*.

